# Supplementary material for: The mIAA7 degron improves auxin-mediated degradation in Caenorhabditis elegans
Source: G3 (Bethesda). 2022 Aug 27;12(10):jkac222. doi: 10.1093/g3journal/jkac222 (PMC9526053; doi:10.1093/g3journal/jkac222)
Supplement: jkac222_Supplemental_Figure_S1 [file jkac222_supplemental_figure_s1.pdf]

IAA proteins

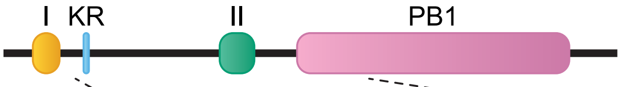


```
IAA17 71-114 AID*   KRGFSETVDLKLNLNNEPANKEGSTTHDVVTFDSKEKSACPKDPAKPPAKAQVVGWPPV
IAA17 65-132 mAID   KRGFSETVDLKLNLNNEPANKEGSTTHDVVTFDSKEKSACPKDPAKPPAKAQVVGWPPV
IAA7 37-104 mIAA7   KRGFSETVDLMLNLQSN---KEGSVDLKNVSAVPK-EKTTLKDPSKPPAKAQVVGWPPV
                    ********** ***:.:   ****.  . *:   * :.:  ***:**************

IAA17 71-114 AID*   RSYRKNVMVSCQKSS--------------GGPEAAAFVKVSMDGAPYLRKIDLRMYKSY
IAA17 65-132 mAID   RSYRKNVMVSCQKSS--------------GGPEAAAFVKVSMDGAPYLRKIDLRMYKSY
IAA7 37-104 mIAA7   RNYRKNMMTQQKTSSGAEEASSEKAGNFGGGAAGAGLVKVSMDGAPYLRKVDLKMYKSY
                    *.****:*.. :.**              **  .*.:*************:**:*****
```
