# Supplementary figures and images for: The mIAA7 degron improves auxin-mediated degradation in Caenorhabditis elegans
Source: G3 (Bethesda). 2022 Aug 27;12(10):jkac222. doi: 10.1093/g3journal/jkac222 (PMC9526053; doi:10.1093/g3journal/jkac222)

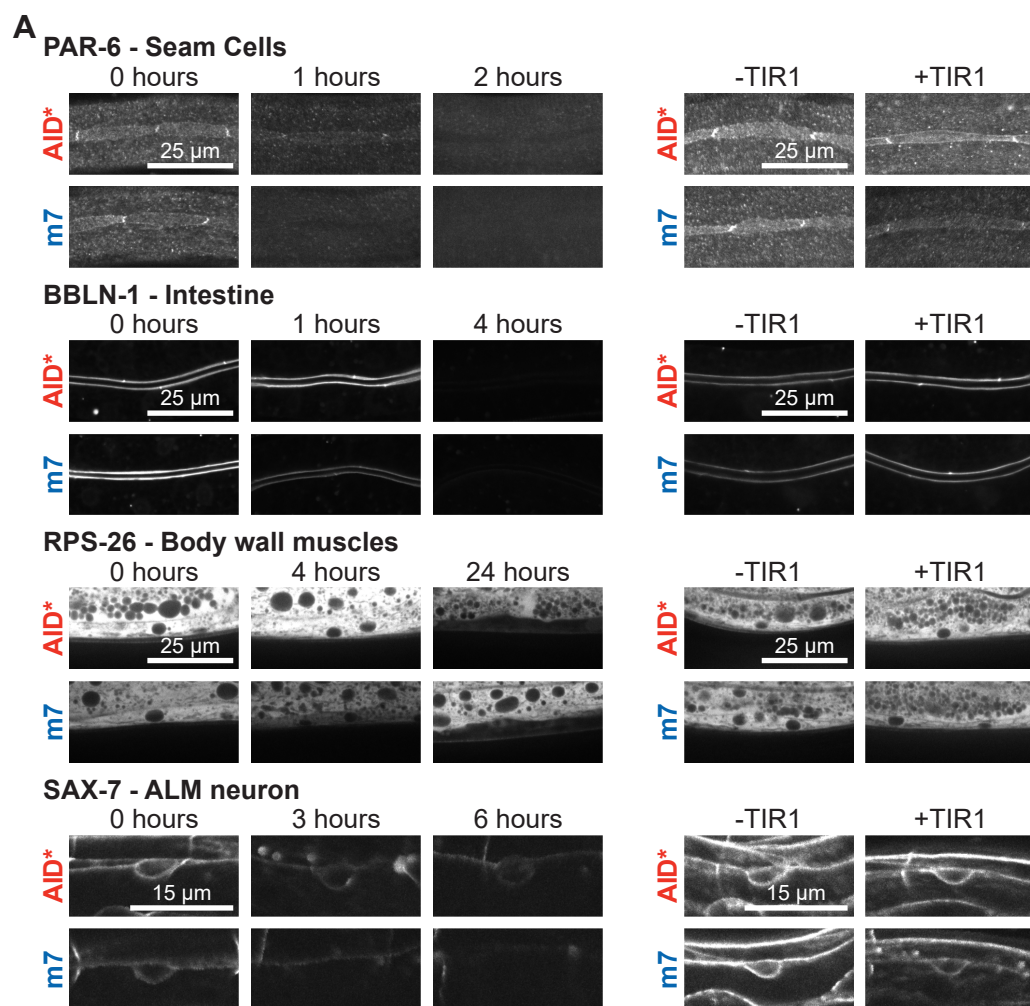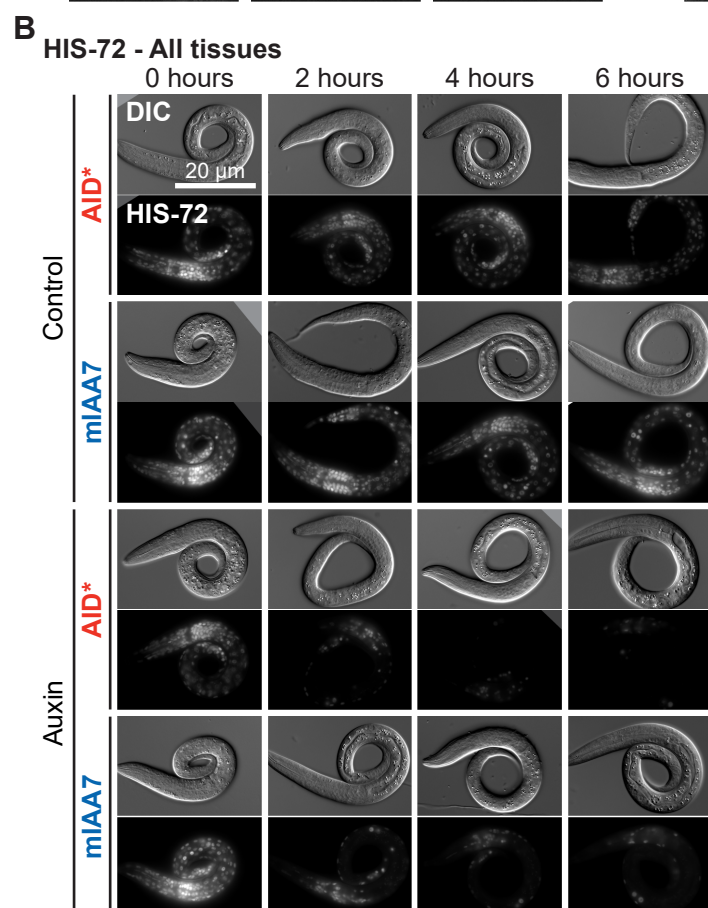

Supplement: jkac222_Supplemental_Figure_S2 [file jkac222_supplemental_figure_s2.pdf]

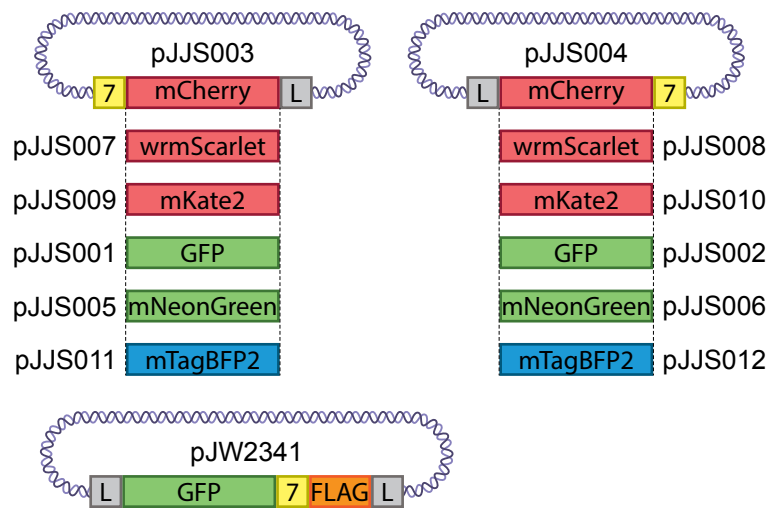

Supplement: jkac222_Supplemental_Figure_S3 [file jkac222_supplemental_figure_s3.pdf]
