# Supplementary material for: The mIAA7 degron improves auxin-mediated degradation in Caenorhabditis elegans
Source: G3 (Bethesda). 2022 Aug 27;12(10):jkac222. doi: 10.1093/g3journal/jkac222 (PMC9526053; doi:10.1093/g3journal/jkac222)
Supplement: jkac222_Supplemental_Figure_Legends [file jkac222_supplemental_figure_legends.docx]

**Figure S1 – Schematic overview of the IAA proteins and the sequences of the different AID degrons that have been derived from them.** Amino acids in bold are part of the degron sequences. IAA = Indole-3-acetic acid; AID = auxin inducible degron; I = domain I; KR = conserved lysine and arginine residue; II = domain II; PB1 = Phox and Bem1p domain.

**Figure S2** **– The mIAA7 degron robustly increases the efficiency of AID-induced protein degradation for several proteins across multiple tissues and cellular compartments.** **(A)** Comparison between AID*- and mIAA7-mediated degradation for indicated proteins and tissues. PAR-6 was measured at the apical domain of seam cells in L2 larvae on 5 μM auxin, BBLN-1 was measured at the apical domain in the intestine of L3 larvae treated with 50 μM auxin, SAX-7 was measured at the plasma membrane in the ALM neuron cell body of L3 larvae treated with 5 μM auxin, and RPS-26 was measured in the cytoplasm of body wall muscles of L2 larvae treated with 1 mM auxin. m7 = mIAA7 **(B)** Comparison between AID*- and mIAA7-mediated degradation for HIS-72 in all tissues of synchronized control or 4 mM auxin-treated L1 larvae. Images shown are representative maximum intensity projections that were acquired and displayed with the same settings for comparison, except for RPS-26 and HIS-72 for which a single plane is presented.

**Figure S3 – Schematic overview of the mIAA7 repair template plasmids.** 7 = mIAA7 degron; L = linker sequence; GFP = green fluorescent protein; BFP = blue fluorescent protein; FLAG = 3x FLAG-tag.
